# Clinicopathological Features of Hereditary Nephritis in the Iranian Population: Analysis of a 14-Year Survey in Kidney Biopsies From a Large Referral Center

**DOI:** 10.34172/aim.2024.02

**Published:** 2024-01-01

**Authors:** Amir Emami, Fatemeh Nili, Maryam Sotoudeh Anvari, Samaneh Salarvand, Golnar Seirafi

**Affiliations:** ^1^Department of Pathology, Imam Khomeini Hospital Complex, Tehran University of Medical Sciences, Tehran, Iran; ^2^Department of Molecular Pathology, Children Medical Center, Tehran University of Medical Sciences, Tehran, Iran; ^3^Tehran University of Medical Sciences, Tehran, Iran

**Keywords:** Alport syndrome, Hereditary nephritis, Thin basement membrane nephropathy

## Abstract

**Background:**

Hereditary nephritis (HN), including Alport syndrome (AS) and thin basement membrane nephropathy (TBMN), is a rare genetic cause of hematuria. A definitive diagnosis requires electron microscopy (EM). Therefore, the clinical characteristics of these conditions are less known. This study aimed to determine the percentage and clinicopathological features of HN in patients from a referral center in Iran.

**Methods:**

We checked kidney biopsy reports from 2007 to 2021 and extracted cases with HN. Fresh specimens of the cases diagnosed in the last two years were stained by immunofluorescence (IF) for collagen type IV alpha chains. EM findings in these cases were re-evaluated and categorized as diffuse glomerular basement membrane (GBM) thinning, definite, and suspicious features of AS.

**Results:**

We analyzed 3884 pathology reports of kidney biopsies from 2007 to 2021 and identified 210 patients (5.4%) with HN, with a mean age of 13.78±12.42 years old. Hematuria with proteinuria (53.3%), isolated hematuria (44.2%), and proteinuria with hematuria and increased creatinine (2.5%) were found in these patients. The re-evaluation of EM findings revealed GBM thinning, definite, and suspicious findings of AS in 37.5%, 43.8%, and 18.8% cases, respectively. The most common diagnosis in 32 cases after the IF study was X-linked AS (71.9%), and 6.2% of cases were autosomal recessive AS. TBMN and autosomal dominant AS remained the differential diagnoses in 21.9%.

**Conclusion:**

It was found that EM is helpful for the primary diagnosis of patients with definite AS. Immunostaining improves the diagnostic sensitivity for the differentiation of those with suspicious EM findings and determines the inheritance pattern. However, a multidisciplinary approach for a subset of cases is required for the best diagnosis and management.

## Introduction

 Alport syndrome (AS, OMIM#301050) and thin basement membrane nephropathy (TBMN, OMIM#141200) are heterogeneous genetic conditions characterized by structural abnormalities in the glomerular basement membrane (GBM).^1^ AS is associated with hematuria, increased proteinuria, and advanced renal failure that varies with age.^2^ It is caused by a genetic defect in type IV collagen, with a prevalence of about one in 5000 for the gene mutation and one in 10 000 for the disease.^2^ Approximately 85% of AS cases are caused by mutations in the *COL4A5* gene on the X chromosome,^3^ while other genetic causes involve the *COL4A3/COL4A4* mutation on chromosome 2, which cause a defect in the α3 and α4 chains of type IV collagen and are transmitted as an autosomal recessive or rarely an autosomal dominant disease.^3,4^ Unlike X-linked cases, which mostly lead to end-stage renal disease (ESRD) in men, autosomal cases can cause a variety of symptoms, ranging from hematuria to ESRD in men or women.^5,6^

 TBMN, also known as benign familial hematuria, affects about 1% of the general population.^7-9^ Approximately 50% of patients with TBMN present as an autosomal dominant pattern, and 40–50% as mutations in the *COL4A3* or *COL4A4* genes on the X chromosome.^10^ These mutations cause slight reductions in the length of the α3α4α5 network in collagen type IV of the GBM, unlike AS, where type IV collagen networks are destroyed or deformed severely.^7,11^ Nevertheless, about 40% of TBMN cases are carriers for autosomal recessive AS with defects in the α3 and α4 chains. In the biopsy specimen of these patients, the GBM is usually less than 250 nm on EM, while in AS, the diagnostic features in EM are multilayering of lamina densa, ruptured areas, membrane inclusions, and irregularities in the subepithelial surface.

 It is important to differentiate classic TBMN from AS. Unlike TBMN, which is a benign disease, the prognosis for AS is poor.^1,12^

 The EM study is the first way to distinguish AS from TBMN, but it does not differentiate between types of inheritance patterns of AS.^13^ Moreover, in TBMN cases who are carriers for the AS, or early stages of AS, thinness of the GBM might be the only finding in EM, making it impossible to differentiate these diseases from each other.^14^

 The aim of this study was to determine the prevalence, clinical, and demographic status of hereditary nephritis (HN) in patients from a referral center in Iran, compared with other diagnoses. Additionally, it investigated the potential value of immunostaining for the alpha chains of collagen type IV for better diagnosis in these patients, providing valuable diagnostic information for the identification and characterization of HN.

## Materials and Methods

###  Patient Selection, Demographic, and Pathologic Data Collection

 We conducted a retrospective analysis of 3884 cases in the Pathology Department of the Imam Khomeini Hospital Complex (IKHC) of Tehran University of Medical Sciences from March 2007 to March 2021. The study aimed to investigate the clinical and demographic status of HN in patients referred to the center compared with other diagnoses.

**Figure 1:**
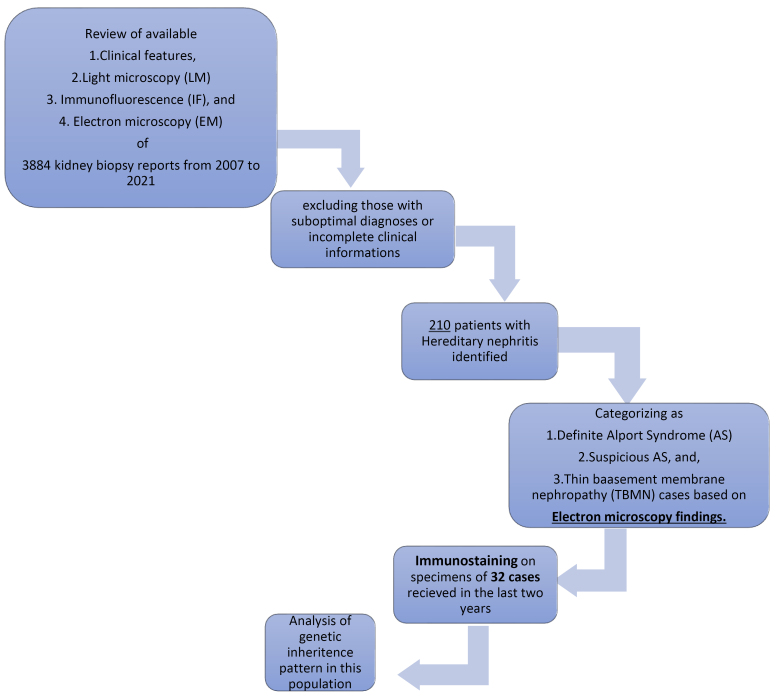
The Flow Diagram Showing Participant Progression Throughout the Study

 All the light microscopy (LM), immunofluorescence (IF), and electron microscopy (EM) reports of the kidney biopsy samples, along with available clinical and demographic data, including age, gender, and clinical manifestation of referred cases to the IKHC of Tehran University of Medical Sciences from March 2007 to March 2021, were reviewed, and cases with HN (AS, suspicious for AS, and TBMN) were identified based on the combination of clinical, LM, IF, and EM data. Clinical data were extracted from recorded files in the pathology department and telephone calls in cases of incomplete information. Cases with non-diagnostic specimens and those with incomplete information were excluded from specific analyses. Figure 1 shows participant progression throughout the study.

###  Electron Microscope Study

**Figure 2:**
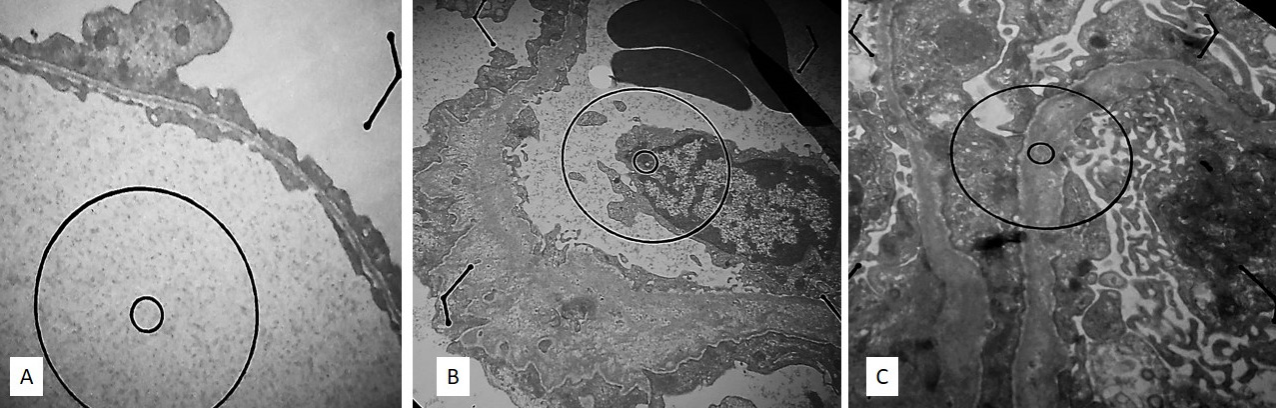
Electron Microscopy Features in the Case of **(A)** TBMN, **(B)** definite AS, and **(C)** Suspicious AS

 The ultrathin sections on prepared grids for EM of cases with HN were retrieved from the pathology archive and reviewed by nephropathologists under EM (Philips 208s, the Netherlands). Based on EM findings, HN cases were categorized as follows:

1. Definitive AS: The presence of diffuse GBM abnormalities, including alternating thick and thin segments, multilayering of the lamina densa, basket weave appearance, and scalloping on the subepithelial surface
2. Suspicious for AS: The presence of diffuse GBM thinning and focal thickening with splitting or multilayering of lamina densa
3. Diffuse glomerular basement membrane thinning: Comparing the average GBM thickness in different areas of the glomerulus with reference intervals (Figure 2).

###  Immunofluorescence for Alpha Chains of Collagen Type IV

 As discussed in the introduction, more investigations, including the IF study for alpha chains of collagen type IV on fresh specimens, are required for the distinction of TBMN and AS. Accordingly, during the last two years of the study (2019‒2021), an immunofluorescence study for alpha chains of collagen type IV was also performed on fresh specimens of newly diagnosed cases of HN.

**Figure 3:**
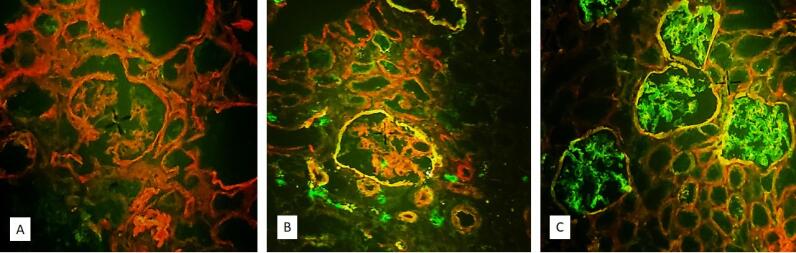
Immunofluorescence Staining in a Case of **(A)** X-linked AS (the Absence of COL4A5 in GBM and Bowman Capsule), **(B)** ARAS (the Absence of Staining in GBM but Preserved Staining in Bowman Capsule), and **(C)** Normal Staining in TBMN, ADAS, or Subset of XLAS

 After preparing suitable 4-micron sections with a frozen section machine, staining was performed using a fluorochrome-conjugated anti-collagen IV cocktail (COSMO BIO Company, Japan). For this purpose, 20 μL of the solution was placed on tissue slices and incubated for 30 minutes at room temperature. Then, the slices were washed with phosphate-buffered saline, mounted with a special adhesive, and evaluated under an immunofluorescence microscope (Zeiss, Germany). An X-linked AS diagnosis was made if the GBM was only stained red, while orange or green staining of the GBM was considered normal. The presence of the mosaic pattern indicated the carrier, and weak staining was considered TBMN. Autosomal dominant or recessive AS was diagnosed if red staining was only detected in the GBM of the proximal tubule, but green and orange staining was observed in the Bowman’s capsule (Figure 3).

###  Statistical Analysis

 Data were analyzed using the Statistical Package for Social Sciences (SPSS) software, version 23 (IBM, Armonk, New York). Categorical variables were reported using frequencies and percentages and were compared between groups using the chi-square test. Continuous variables were compared between groups using the one-way analysis of variance test. The Kappa agreement coefficient was used to determine the agreement between the two diagnostic methods. The level of statistical significance was considered to be *P*< 0.05.

## Results

###  General Demographic Data

 Among 3884 cases with kidney biopsy reports, based on the combination of clinical findings, LM, IF, and EM data, HN was diagnosed in 210 (5.4%) cases. From these 210 cases, 120 (57.1%) patients were male and 90 (42.8%) were female (male: female ratio = 1.3). The mean age of the patients was 13.78 (9.9‒18.2, ranging between 2 and 54 years old). The peak age for the diagnosis of HN was 10‒20 years old.

 The most common clinical findings among the 210 patients were hematuria with proteinuria (53.3%), followed by isolated hematuria (44.2%) and proteinuria in association with hematuria, and renal failure (2.5%). Reviewing the hematoxylin and eosin slides of these 210 cases on light microscopy revealed that most of the cases [188 out of 210 (89.5%)] had minimal histopathological findings, and 22 samples (10.5%) had focal and segmental glomerulosclerosis with a variable percentage of tubular atrophy and interstitial fibrosis. Interstitial infiltration of foam cells was also noted in 62% of specimens.

 Further evaluations of hearing and visual disorders, as well as family history, was only observable in 100 patients with AS. Based on the results, 57 and 14 cases suffered from hearing and visual disorders, respectively. A family history of renal disease was also reported in 62 (62%) patients, which was associated with hearing disorders and visual problems in 15 (15%) cases (Table 1).

**Table 1:** Age and Gender Distribution, as Well as Clinical LM and EM Findings on 3674 Cases Without and 210 Cases With Hereditary Nephritis

**Population (N)**	**Cases With Kidney Biopsy Without Hereditary Nephritis (3674)**	**Cases With Hereditary Nephritis (210)**
Age (mean ± SD)	29.4 ± 19.0	13.78 ± 12.42
Gender (M/F), No. (%)	1947/1727 (53/47)	120/90 (57.2/42.8)
Clinical manifestations, No. (%)		
Proteinuria and hematuria	1433 (39)	112 (53.3)
Hematuria	220 (6)	93 (44.2)
Hematuria + Proteinuria + Renal failure	-	5 (2.5)
Family history of renal failure No. (%)	Not detectable in all patients	62 (62) *
Hearing disorders	Not detectable in all patients	57 (57) *
Primary EM diagnosis, No. (%)		
TBMN	-	30 (14.3)
Definite AS	-	152 (72.4)
Suggestive for AS	-	28 (13.3)
Light microscopy findings		
Minimal histopathological changes	440 (12)	188 (89.5)
Focal and segmental glomerulosclerosis	588 (16)	22 (10.5)
Membranous glomerulopathy	661 (18%)	-

SD: Standard deviation; AS, Alport syndrome; TBMN, thin basement membrane nephropathy; EM: electron microscopy. *From 100 patients.

 Immunofluorescence studies for routine immune markers (i.e., immunoglobulin G, immunoglobulin A, immunoglobulin M [IgM], C3, C4, and C1q) were negative in most of these cases or non-specific deposits of IgM in sclerotic glomeruli.

 Among the remaining 3674 cases, 1433 (39%) patients clinically presented with symptoms of nephrotic syndrome as the most common clinical manifestation. A total of 661 (18%) patients were diagnosed with membranous glomerulopathy, as the most common diagnosis. The mean age of the patients was 29.4 (28.6‒30.1), which was significantly higher than that of cases with HN (*P* < 0.001). Overall, 1947 (53%) patients were male. No significant statistical difference was found in the gender distribution of patients with or without HN (*P* = 0.57, Table 1).

###  Immunofluorescence Findings for Alpha Chains of Collagen Type IV

 Due to budget and time constraints and the availability of fresh samples, we could only conduct an IF study on the last 32 diagnosed cases in the last two years of the study. Based on immunofluorescence evaluations, X-linked AS was identified in 23 out of 32 (71.9%) patients. Two (6.2%) cases were autosomal recessive AS (ARAS). In the remaining 7 (21.9%) patients, TBMN vs. autosomal dominant AS remained differential diagnoses.

 The re-evaluation of the EM findings of these 32 patients revealed definitive features of AS (as previously described) in 14 out of 32 (43.8%) of the patients. In 12 (37.5%) cases, diffuse GBM thinning was the only ultrastructural pathologic finding. In addition, 6 (18.8%) cases represented diffuse GBM thinning in association with the foci of thickening and irregularity, which was diagnostic of HN but suspicious for AS.

 GBM thinning was found in 21.7% of patients with X-linked AS, while none of the patients with ARAS showed GBM thinning. XLAS was confirmed in all cases with suspicious EM findings. Both ARAS cases demonstrated definite features of AS in the EM study (Table 2).

**Table 2:** Gender Distribution, Clinical, and EM Findings in Different Groups of Hereditary Nephritis, Based on Immunofluorescence for the Alpha Chains of Collagen Type IV (Performed on 32 Samples)

**Final Diagnosis, N**	**XLAS**	**ARAS**	**TBMN vs. AS**	***P*** ** value**
Gender				
Male/female	10/13	1/1	4/3	> 0.05
Clinical manifestation				
Isolated hematuria	7 (30%)	0	6 (86%)	> 0.05
Hematuria and proteinuria	15 (65%)	1 (50%)	1 (14%)	
Proteinuria and renal failure	1 (5%)	1 (50%)	0	
EM findings				
Definite for AS	12	2	0	< 0.05
Suspicious for AS	6	0	0	
Diffuse GBM thinning	5	0	7	

AS, Alport syndrome; TBMN, thin basement membrane nephropathy; XLAS: X-linked Alport syndrome; GBM, Glomerular basement membrane; ARAS, autosomal recessive AS.

 Gender distribution and clinical symptoms in different groups of these patients are also provided in Table 2. All cases with diffuse GBM thinning and normal immunostaining revealed isolated hematuria. Proteinuria in association with hematuria was the clinical history of only one case in this group. There was a significant agreement between immunofluorescence and EM findings (kappa = 0.604, *P* = 0.001).

## Discussion

 AS is a rare genetic disorder, involving one in 5000 people.^15^ It comprises 0.5% of newly diagnosed ESRD cases in adults and 12.9% in children.^15^ The clinicopathologic diagnosis of AS is a challenging issue.^16^ A Japanese working group introduced a diagnostic criterion in 2015.^17^ The major primary clinical criteria were persistent hematuria. Mutations in the α chains of collagen type IV genes, abnormal expression of type IV collagen α chains, and GBM-specific abnormalities on EM are defined as secondary criteria. A family history of kidney disease, bilateral sensorineural deafness, ocular abnormalities, and diffuse leiomyomatosis were considered accessory criteria. The patient should fulfill one or more of the secondary or accessory criteria in addition to persistent hematuria.^17^

 Due to limited access to EM, a lack of immunofluorescence the United States Food and Drug Administration-approved commercial kits, and difficulties in establishing or requesting molecular genetic tests, most cases in developing countries may be missed or only diagnosed based on accessory criteria and clinical findings. Here, 5.4% AS and TBMN were reported in all biopsy-proven kidney samples referred to the largest EM center in Iran. The only available data and study from Iran is a recently published study from Shiraz University of Medical Sciences, which reported 22 (0.77%) HN patients among 2865 kidney biopsies in a 16-year survey.^18^ The large difference between the results of these two studies can mainly be explained by the fact that our center, as the only available EM center in Tehran, is a highly selective referral center that receives more suspicious cases for the confirmation of HN and handles at least 30% more kidney biopsy samples (3884 in 12 vs. 2865 in 16 years) per year compared to their center.

 As expected, most of the newly diagnosed patients were children with a mean age of 13.7 years. XLAS was the main population of our cases. Although it more commonly occurs in men, women included a significant population of the cases in our study.

 All the cases had a history of persistent hematuria for more than 3 months as the major criteria. Associated proteinuria and renal failure were observed in about 53.3% and 2.5% of cases, respectively. Clinical symptoms in AS depend on the mode of inheritance. In XLAS, which is the more prevalent form, both proteinuria and hematuria are observed in about 73% of cases.^19^ In male patients, proteinuria is developed at the early stages of the disease in childhood.^20^

 A family history of renal disease was reported in about 62% of our patients. This feature is also expected to be higher in XLAS. There is usually a positive family history of kidney disease in most patients. De novo variants without family history were found in about 15% of the cases.^21^

 Sensorineural hearing loss occurs in men with XLAS later in childhood in about 90% of the patients and develops in only 12% of females by the age of 40. In our study, this problem was detected in 57% of the patients.^22^ The frequency of ocular abnormalities is less, which was identified in 14% of the patients.

 Although the percentage of clinical features in our study is nearly similar to the reported previous data, we think, selection bias in performing kidney biopsy and referring patients with clinically unexplained symptoms has been effective in achieving these results. To determine the true prevalence in the Iranian population, patients or families from different regions of the country, who are clinically suspected but not have been biopsied, should be evaluated and included as well.

 Based on the findings, LM findings are not specific, depending on the stage of the disease, and may be consists of minimal changes or glomerulosclerosis, tubular atrophy, and interstitial fibrosis. Interstitial foam cells in the absence of long-time proteinuria are indicative of AS. The EM changes of GBM, as previously described, are specific for AS, which was identified in 43.8% of the patients. However, these characteristic pathologic findings are identifiable in the form of disease progression, even in males with XLAS.^15^ Incomplete features or only GBM thinning may be observed in females and earlier stages of the disease.^23^ In our study, 18.8% and 37.5% of the patients showed suspicious features of AS and diffuse GBM thinning. The distinction between TBMN and AS is an interesting and challenging issue in this situation.^15^ AS is a progressive disease with hearing and ocular abnormalities, while TBMN is generally a benign disease, which does not progress to ESRD. Immunostaining for α5 chains of collagen type IV can be of some help. Complete loss of α5 is indicative of XLAS.^1^ It occurs in 80% of males, while females exhibit a mosaic pattern due to the inactivation of the X chromosome. Negative staining in GBM but its expression in Bowman capsule is diagnostic of ARAS.^1,15^ In our study, 5 out of 12 cases with GBM thinning were categorized as XLAS after immunostaining. Normal expression makes a challenge, as this pattern may be found in ADAS, TBMN, and 20% of XLAS.^15^ In our study, immunostaining was performed on 32 recently preserved samples. Abnormal expression was detected in 25 patients (23 XLAS and 2 ARAS). Only 7 patients revealed normal expression. As a result, immunostaining improved the diagnostic accuracy of AS from 43.8% to 78.1%. Genetic counselling, patient follow-ups, clinicopathologic correlation, and molecular studies are required for the distinction of TBMN and AS in the remaining 7 (21%) patients. It is well-established that ESRD is never developed in patients with isolated hematuria without proteinuria.^24^ Females with XLAS and individuals with ADAS begin to show proteinuria in 7 years and 17 years, respectively,^15,22,25^ so careful follow-up of the patients is crucial.

 There is no definite treatment for AS. Kashtan et al have recently published a revised recommendation for the clinical practice and management of AS^26^ and recommended the initiation of ACE inhibitor treatment in all men with XLAS and all males and females with ARAS. Females with XLAS and both individuals with ADAS can start the treatment following the onset of micro-albuminuria.^26^

 This study presents the largest report on the clinicopathological features of Iranian patients with HN. Immunostaining was first used to identify the most common patterns of inheritance in a randomly selected group of patients. The results of this study can be valuable for future research and the development of guidelines for the diagnosis and management of Iranian patients with HN.

 However, this study had several limitations. Due to the lack of access to EM and its cost, not all patients or families who were suspicious of HN were evaluated and diagnosed, which may have resulted in an underestimation of the true prevalence of HN in the Iranian population. On the other hand, a potential selection bias by the nephrologists for referring kidney biopsy samples to our center may have influenced our results. Clinical information was retrieved from the recorded files in our department or telephone calls to the patients, which may have been influenced by some errors, including potential inconsistencies or errors in reporting or recording. Finally, we could not validate the diagnosis of our cases by genetic studies. To address these limitations, future multicenter studies involving all suspicious cases from different regions and accurate assessment of clinical findings, along with genetic studies for problematic cases, may provide more information.

## Conclusion

 HN accounts for about 5.4% of biopsy-proven kidney biopsies in our large referral center for EM. Most cases are patients with XLAS. EM is helpful for the diagnosis of most patients with hematuria and definite AS syndrome. Immunostaining has a specific capacity for the distinction of the majority of XLAS and ARAS with only GBM thinning from the ADAS and TBMN. A multidisciplinary approach for the remaining limited cases is required to obtain the best diagnosis and management.
